# Valorized Shrimp Shell-Derived Aerogel for Trace Enrofloxacin Removal from Aquaculture Wastewater: Adsorption Performance and Mechanisms Exploration

**DOI:** 10.3390/gels12030247

**Published:** 2026-03-15

**Authors:** Chengci Liu, Lei Huang, Sihan Wei, Bohao Qi, Jinhua Xu, Xiaodong Xu, Lu Qiao, Zhen Yang, Yuanyuan Ren, Jincheng Li, Yingchun Mu, Mutai Bao, Meitong Li, Zhiyang Zhao, Xin Hu

**Affiliations:** 1 Tianjin Key Laboratory of Organic Solar Cells and Photochemical Conversion, College of Chemistry and Chemical Engineering, Tianjin University of Technology, Tianjin 300384, China; chengci1223@stud.tjut.edu.cn (C.L.); huanglei@tjut.edu.cn (L.H.); yj20251014@stud.tjut.edu.cn (S.W.); tjutlmt@email.tjut.edu.cn (M.L.); 2 Chinese Academy of Fishery Sciences, Beijing 100141, China; qibohao@cafs.ac.cn (B.Q.); xujh@cafs.ac.cn (J.X.); xuxiaodong@cafs.ac.cn (X.X.); qiaolu@cafs.ac.cn (L.Q.); yangzhen@cafs.ac.cn (Z.Y.); renyuany@cafs.ac.cn (Y.R.); lijc@cafs.ac.cn (J.L.); muyc@cafs.ac.cn (Y.M.); 3 Hainan Fisheries Innovation Research Institute, Chinese Academy of Fishery Sciences, Sanya 572000, China; 4 Key Laboratory of Marine Chemistry Theory and Technology Ministry of Education, Frontiers Science Center for Deep Ocean Multispheres and Earth System, Ocean University of China, Qingdao 266100, China; mtbao@ouc.edu.cn; 5 College of Chemistry & Chemical Engineering, Ocean University of China, Qingdao 266100, China; 6 School of Chemistry and Materials Science, Nanjing University of Information Science & Technology, Nanjing 210044, China

**Keywords:** shrimp shell, aerogel, porous material, enrofloxacin, adsorption

## Abstract

Enrofloxacin (ENR), as a widely used antimicrobial agent in aquaculture, poses potential risks to ecosystems and human health due to its environmental persistence. Therefore, it is of great significance to explore efficient methods for removing ENR from aquaculture wastewater. In this study, a series of shrimp shell-derived aerogel (MBC300–MBC700) were fabricated from *Litopenaeus vannamei* shells through chemical modification followed by pyrolysis at 300–700 °C, and their adsorption performance and mechanisms toward ENR were systematically investigated. The modified porous materials exhibited a well-developed micro–mesoporous structure, high specific surface area, and abundant surface functional groups. Meanwhile, MBC400 demonstrated the highest adsorption capacity for ENR, reaching 14.56 mg/g, with a corresponding specific surface area of 77.71 m^2^/g. The adsorption kinetics followed the pseudo-second-order model, and the isothermal data were better fitted by the Freundlich model, indicating a chemisorption-dominated, heterogeneous multilayer adsorption process. Thermodynamic analysis revealed that the adsorption was spontaneous (ΔG < 0) and endothermic (ΔH > 0). In regeneration experiments, 30% ethanol solution achieved the best desorption efficiency for MBC400, with adsorption efficiency remaining above 75% after three cycles. Based on the characterization and adsorption results, adsorption mechanism of ENR on MBC400 was elucidated as a synergistic effect of hydrogen bonding, π–π stacking, electrostatic interaction, and surface complexation. This study provides a novel strategy and theoretical basis for the high-value utilization of shrimp shell waste and for the efficient removal of fluoroquinolone antibiotics from aquaculture effluents.

## 1. Introduction

Antibiotics are the most widely used in aquaculture for eradicating bacterial diseases. It was typically administered at therapeutic doses below 0.2 g/kg to improve feed utilization and survival rate [1]. However, prolonged and extensive antibiotics use leads to residues in water, sediments, and aquatic organisms, resulting in various adverse environmental and health effects. Enrofloxacin (ENR), a third-generation fluoroquinolone antimicrobial agent, is extensively used in Chinese aquaculture due to its potent antibacterial activity, broad spectrum, high bioavailability, long half-life, and low cost [2]. According to the China Fishery Statistical Yearbook (2025), China’s annual aquaculture production exceeds 70 million tons, with crustaceans such as *Litopenaeus vannamei*, accounted for 15% of this output. The annual usage of ENR is approximately 5000 tons, primarily for treating common diseases such as gill rot, enteritis, and aesculapian disease [3]. Nevertheless, as only 20–30% of antibiotics are metabolized by organisms, the majority of antibiotics are excreted as active compounds into the environment, with reported excretion rates ranging from 20.0 to 97.0% [4]. The chronic exposure to ENR (>100 μg/kg) may impair cartilage development and reproductive function in aquatic organisms. Therefore, strict dosage control and compliance with withdrawal periods are essential to safeguard product quality and consumer safety.

In addition to controlling the source of antibiotic usage, the efficient removal of existing antibiotic residues from aquatic environments is equally critical. Currently, various antibiotic-removal technologies have been developed worldwide, including biological treatments [5], advanced oxidation processes [6], chemical precipitation [7], reverse osmosis [8], membrane separation [9], and adsorption [10]. Among these, the adsorption method stands out as a preferred technique for removing antibiotics because of its operational simplicity, cost-effectiveness, reliable treatment efficiency, and minimal secondary pollution. Carbon-based adsorbents such as aerogel and activated carbon have attracted widespread attention in both academic and industrial applications owing to their low production cost, favourable environmental compatibility, and potential for carbon sequestration. Current research on aerogel feedstocks predominantly focuses on lignocellulosic biomass such as rice straw, sawdust, tea residue, microalgae, and corn straw [11]. To further promote the resource utilization of solid waste, recent studies have increasingly explored the preparation of functional aerogel from marine organic-rich wastes [12].

As major by-products of the seafood processing industry, shrimp shells are not only rich in organic components, but also readily retain oxygen-containing functional groups after pyrolysis, endowing them with inherent advantages for high-performance adsorbents [13]. As the world’s largest producer and processor of crustacean products, China produced a total of 7.8 million tons of shrimps and crabs in 2025. The shrimp shell account for 35–40% of the total weight, corresponding to an annual discharge exceeding 1.78 million tons [14]. Currently, approximately 70% of these by-products are landfilled or incinerated, which not only wastes valuable resources such as chitin, protein, and calcium carbonate, but also generates environmental burdens [15]. Shrimp and crab shells produce aerogel with organic–inorganic composite matrices that enhance adsorption of polar organic pollutants, thereby enabling immobilization of processing waste [16]. Compared with commercial activated carbon, shrimp shell aerogel reduces production costs by 40–55% and typically requires minimal pretreatment, aligning with circular-economy principles [17]. The on-site conversion of such waste into aerogel for pollutant treatment could synergistically integrate solid-waste with in situ pollution control in aquaculture systems. However, systematic investigations on the application of shrimp shell aerogel for antibiotic removal remain relatively limited.

This study aims to address the dual challenges of ENR residue pollution and the resource utilization of shrimp shell waste by developing efficient shrimp shell-derived aerogel adsorbents for removing ENR from aquaculture water. For the first time, this study utilized *Litopenaeus vannamei* shrimp shells as raw material to prepare aerogel for ENR adsorption. A series of shrimp shell-derived aerogel (MBC300–MBC700) was successfully prepared through chemical modification assisted by high-temperature pyrolysis technology. And its adsorption behaviour and mechanism for ENR were comprehensively and systematically investigated. Morphological features, functional groups, specific surface area, and elemental composition of aerogel were characterized using scanning electron microscopy (SEM), Fourier transform infrared spectroscopy (FTIR), Brunauer Emmett and Teller (BET) model, and X-ray photoelectron spectroscopy (XPS). A series of batch experiments was conducted to systematically assess the influence of adsorbent dosage, solution pH, temperature, and initial ENR concentration on the adsorption efficiency. Adsorption mechanisms were elucidated through kinetic studies, adsorption isotherm analysis, and thermodynamic assessment. The regenerative capacity of aerogel was validated through three adsorption–desorption cycles. This study pioneers the conversion of shell waste into a highly efficient adsorbent, providing technical support for the effective removal of fluoroquinolone antibiotics from aquaculture wastewater.

## 2. Results and Discussion

### 2.1. Aerogel Characterization

#### 2.1.1. The Morphological Features

**Figure 1:**
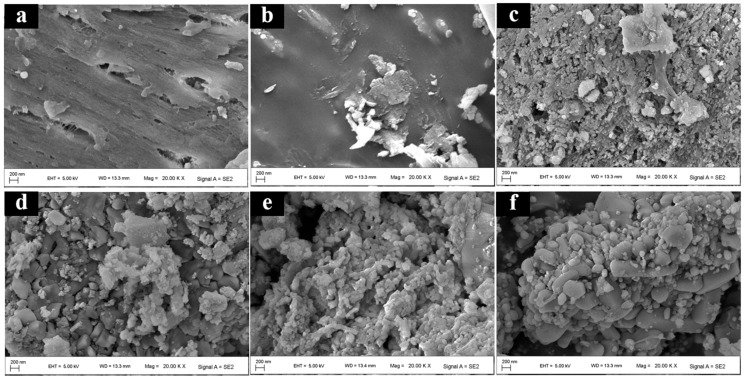
SEM images of aerogel. MBC (**a**); MBC300 (**b**); MBC400 (**c**); MBC500 (**d**); MBC600 (**e**); MBC700 (**f**).

Figure 1 presents SEM images of MBC and its derivatives calcined at different temperatures ranging from 300 to 700 °C (MBC300–MBC700), demonstrating pronounced temperature-dependent changes in surface morphology. As shown in Figure 1a, MBC displayed a relatively regular texture and structure, featuring natural pores and microstructural details. The initial structural degradation became apparent with increasing calcination temperature. Figure 1b exhibited partial decomposition and collapse of the material, accompanied by altered surface morphology. These alterations were attributed to the thermal decomposition of labile organic components at lower temperatures, leading to a restructuring of the surface morphology [18]. Further structural fragmentation and reorganization are observed in Figure 1c. The continued decomposition of organic matter and initial transformation of inorganic components produced evolving pore characteristics and altered particle aggregation.

The increasing calcination temperature induced progressive carbonization of the shrimp shell and crystallization of inorganic phases (From Figure 1d–f). Surface morphology evolved from a complex intermediate state to a more stable high-temperature form. Systematic changes in particle aggregation and pore structure are evident, reflecting the temperature-dependent evolution of the material [19]. The elevated temperatures promoted crystal growth in inorganic phases and enhanced the structural ordering of carbon, producing distinct microstructural features that correlate strongly with calcination temperature [20]. The aerogel prepared from *Litopenaeus vannamei* exhibited a course, porous, and loosely aggregated morphology with irregular particles. Its abundant pore structure enhanced specific surface area, thereby facilitating the ENR adsorption. Consistent with the works of Barszcz et al., thermal activation increased the aerogel’s surface area and enhanced its pore structure [21].

#### 2.1.2. Surface Area

Nitrogen adsorption–desorption isotherms measured at low temperature were used to determine the specific surface area, total pore volume, and pore size distribution of shrimp shell calcined at various temperatures (0, 300, 400, 500, 600, and 700 °C). As illustrated in Figure S1a–f, the isotherm shaped strongly depend on calcination temperature. The isotherm of shrimp shell displayed a relatively flat profile in the low relative pressure (P/P_0_) region (Figure S1a). With increasing calcination temperature (≥300 °C), adsorption “knee” became increasingly distinct and extended, suggesting the progressive development of microporosity (Figure S1b). According to the International Union of Pure and Applied Chemistry (IUPAC) classification of physisorption isotherms [22], isotherms evolved from a Type I shape, indicating a predominantly microporous structure at lower temperatures. Then, it formed a composite profile accompanied by hysteresis loops at higher relative pressures. The appearance of hysteresis loops was indicative of the formation and development of mesopores [23]. For MBC300–MBC700, hysteresis behaviour was consistent with the presence of slit-shaped mesopores, confirming that shrimp shell materials developed a micro–mesoporous composite architecture after calcination between 300 °C and 700 °C. These temperature-dependent changes in micropore and mesopore abundance and structure likely result from thermal decomposition of organic components, phase transformations of inorganic constituents, and structural rearrangement during heat treatment [24].

The specific surface area (A_BET_) was calculated from N_2_ adsorption data using BET equation over the relative pressure range (0.05 < P/P_0_ < 0.20), which was chosen based on adsorption behaviour of shrimp shell-derived aerogel. As summarized in Table S3, BET of the different aerogel exhibited a pronounced dependence on temperature. MBC had a surface area of 4.3605 m^2^/g, which increased to 23.6353 m^2^/g for MBC300 and rose markedly to 77.7090 m^2^/g for MBC400. Beyond this temperature, surface area progressively decreased for MBC500 (62.4027 m^2^/g), MBC600 (57.2715 m^2^/g), and MBC700 (52.1045 m^2^/g), indicating that the maximum surface area was achieved at 400 °C. Similarly, the total pore volume ($V_{total}$) evaluated from the N_2_ adsorbed at P/P_0_ = 0.99, followed a trend analogous to that of A_BET_. MBC400 exhibited the highest value (0.1972 cm^3^/g). These results supported that calcination at 400 °C optimized pore development in the shrimp shell system.

Pore size distributions were derived from the logarithmic plots of adsorption isotherms (Figure S2a–f). MBC exhibited a relatively underdeveloped pore structure. With increasing temperature up to 300 °C (Figure S2b) and 400 °C (Figure S2c), formation and expansion of both micropores and mesopores were evident. MBC400 displayed high uptake at low relative pressures and an isotherm shape consistent with its maximal surface area and pore volume, indicating a well-developed micro–mesoporous composite structure. Annealing at temperatures ≥ 500 °C induced particle coalescence and partial structural collapse (Figure S2d–f), while it diminished the specific surface area and pore volume. The calculated average pore sizes (e.g., 12.96 nm for MBC700; Table S3) were consistent with the structural evolution inferred from the isotherms and pore size distributions. Overall, these results demonstrated that calcination temperature critically controls pore architecture such as specific surface area, pore volume, and pore size distribution of shrimp shell-derived aerogel. Calcination near 400 °C optimized pore development, whereas higher temperatures induced sintering and structural degradation [25].

**Figure 2:**
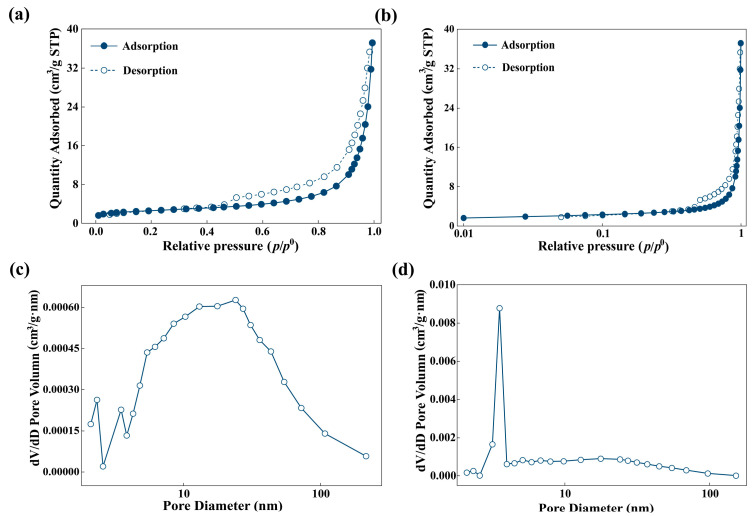
BET analysis of MBC400. Isotherm linear plot (**a**); isotherm log plot (**b**); BJH adsorption dV/dD pore volume (**c**); BJH desorption dV/dD pore volume (**d**).

As shown in Figure 2a,b, nitrogen adsorption isotherm of MBC400 combined Type I and Type IV characteristics, featuring a gradual uptake at low P/P_0_ due to micropore filling and increasing at higher pressures associated with capillary condensation in mesopores. These observations indicated a hierarchical pore structure composed of both micropores and mesopores. BJH pore size distribution curves derived from the adsorption branch displayed a prominent peak in mesopore range (2–50 nm) (Figure 2c), suggesting that calcination at 400 °C facilitated the formation of abundant mesopores. The desorption branch exhibited a sharp peak with a narrow pore size range (Figure 2d), which can be attributed to the “ink–bottle” effect and specific pore connectivity typical of mesoporous materials [26]. Overall, these results confirmed that MBC400 possessed a composite micro–mesoporous structure with a predominance of mesopores, which provided a structural foundation for its subsequent adsorption performance.

#### 2.1.3. Thermochemical Property Analysis

**Figure 3:**
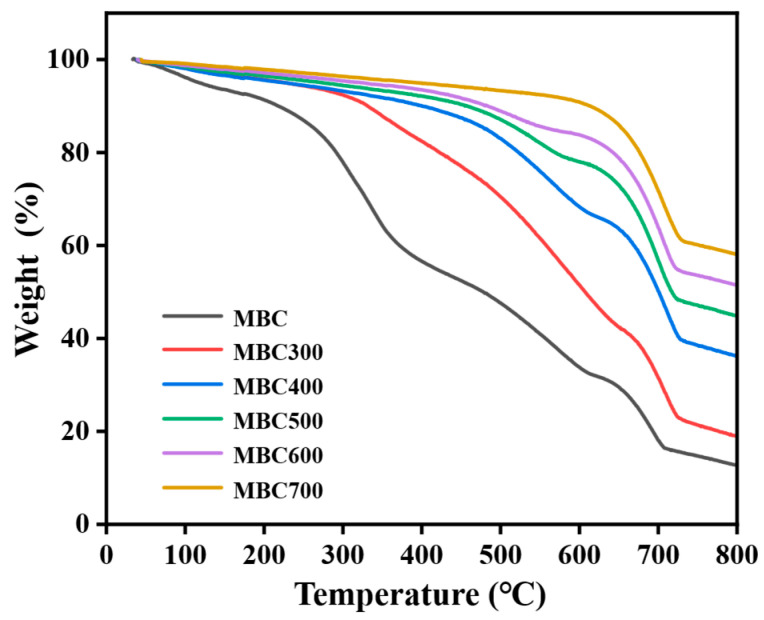
TGA curves of MBC and MBC300–MBC700.

TG curves of MBC and MBC300–MBC700 are shown in Figure 3. The total mass losses for MBC and MBC300–MBC700 were approximately 87.28, 81.02, 63.79, 55.12, 48.51, and 41.88 wt%, respectively. As calcination temperature increased from 300 to 700 °C, the total mass loss gradually decreased. This trend indicates that high-temperature calcination facilitated the early removal of labile decomposable components while enriching thermally stable constituents [27]. Mass loss in all samples was attributed to desorption and evaporation of surface-adsorbed and loosely bound water below 200 °C [28]. In the range of 200–600 °C, MBC exhibited significant mass loss, reflecting pyrolysis of abundant organic constituents such as cellulose and proteins [29]. In contrast, the mass loss of MBC300–MBC700 was mainly associated with decomposition of residual organic matter and elimination of functional groups [30]. As calcination temperature increased, mass loss in this interval decreased considerably. For instance, MBC700 had undergone extensive pyrolysis and contained minimal residual decomposable components. Mass loss plateaued above 600 °C, reflecting the decomposition or crystal phase transformation of inorganic compounds such as CaCO_3_ [31]. In summary, calcination temperature significantly influenced the thermal degradation behaviour by modulating the residual organic matter content and stabilizing the inorganic phases. Based on their pore structure characteristics and thermal stability analysis results, MBC400 was selected as the target adsorbent for subsequent batch adsorption experiments.

#### 2.1.4. The Functional Groups

**Figure 4:**
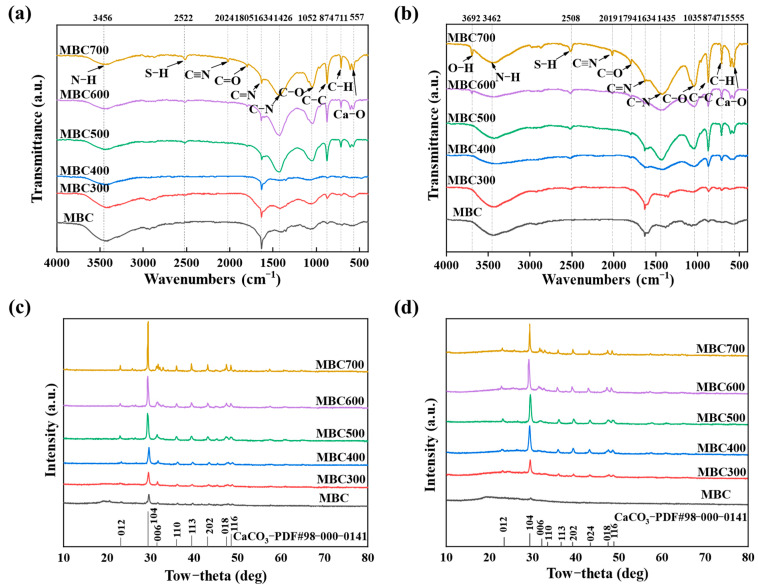
FTIR spectra of shrimp shell-derived aerogel before (**a**) and after ENR adsorption (**b**). XRD patterns of shrimp shell-derived aerogel before (**c**) and after ENR adsorption (**d**).

Figure 4a,b shows the FTIR spectra of shrimp shell-derived aerogel (MBC and MBC300–MBC700). MBC and MBC300–MBC700 exhibited systematic variations in functional groups across the FTIR wavenumber ranges. The broad O–H/N–H band near 3450 cm^–1^ shifted to higher wavenumber and a new peak emerged at 3607 cm^–1^, consistent with enhanced hydrogen-bonding interactions between ENR (−NH/−OH) and aerogel surface groups [32]. The C=O band at 1634 cm^–1^ decreased in intensity, while the ester carbonyl band shifted from 1805 cm^–1^ to 1794 cm^–1^, suggesting electrostatic or hydrogen bonding interactions between carboxyl groups of ENR and shrimp shell-derived aerogel [33]. A band at 2024 cm^–1^ (assigned to C≡N or C≡C) shifted to 2019 cm^–1^, reflecting π–π stacking or coordination interactions. The C–O stretching band that shifted from 1052 to 1035 cm^–1^ indicated involvement of oxygen-containing surface groups in the adsorption process [34]. As calcination temperature increased, several bands change in intensity, demonstrating decomposition and reconstruction of surface functional groups during high-temperature pyrolysis. In particular, MBC700 exhibited more distinct characteristic peaks of C=C, consistent with partial graphitization [35]. These changes collectively confirmed that ENR was adsorbed onto the shrimp shell-derived aerogel via hydrogen bonding, electrostatic interactions, and π–π interactions [36]. The porous structure and modified functional groups provide the main adsorption sites.

#### 2.1.5. XRD

Figure 4c,d present the XRD patterns of shrimp shell-derived aerogel. All samples exhibited a characteristic diffraction peak at 2θ ≈ 29.4°, corresponding to calcite (CaCO_3_, ICDD PDF#98–000–0141), confirming that calcium carbonate was the predominant inorganic phase [37]. As calcination temperature increased (from 300 to 700 °C), peaks’ intensity increased and the full width at half maximum (FWHM) decreased, indicating enhanced crystallinity of CaCO_3_ at higher temperatures. By contrast, MBC displayed a broadened diffraction peak, reflecting its low crystallinity [38]. Furthermore, the crystal structure of shrimp shell-derived aerogel underwent significant changes after ENR adsorption. The intensity of 002 crystal plane diffraction peak (2θ ≈ 26°) decreased and broadened, suggesting the intercalation of ENR molecules into the graphitic microcrystalline interlayers and leading to an increase in interlayer spacing and a reduction in structural ordering [39]. The altered morphology of 100 crystal plane (2θ ≈ 43°) reflected interactions between ENR and oxygen-containing functional groups on aerogel surface, distorting the hexagonal ring planes. Variations in scattering intensity at low-angle region (2θ = 10–20°) suggested the filling of mesopores by ENR molecules [40]. Collectively, these observations support a dual adsorption mechanism. The former primarily disturbed the graphitic microcrystalline structure, while the latter affected the surface functional groups. These differences originated from the specific interactions between ENR molecules and the multi-scale structural features of shrimp shell-derived aerogel [41].

#### 2.1.6. XPS

**Figure 5:**
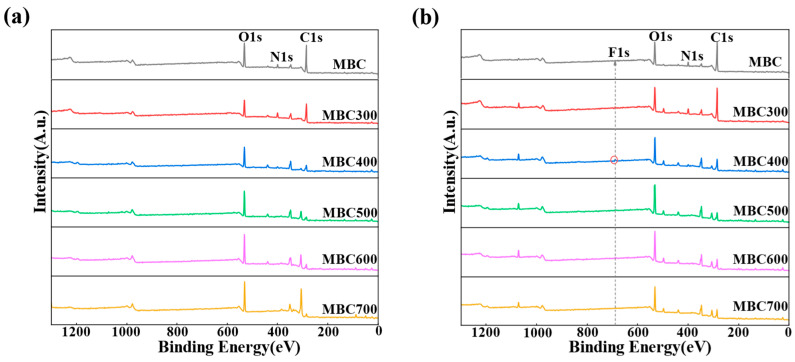
XPS full scan spectra of MBC–MBC700 before (**a**) and after (**b**) ENR adsorption. The red circle marks the characteristic peak position of F1s at approximately 686 eV, and the dashed arrow indicates the spectral intensity variation trend of each sample (MBC300 to MBC700) at the corresponding binding energy.

XPS analysis was conducted to elucidate the surface elemental composition and adsorption mechanisms of ENR on shrimp shell-derived aerogel. As shown in Figure 5a, all samples exhibited characteristic peaks corresponding to C1s at approximately 285 eV, N1s around 400 eV, and O1s near 532 eV before adsorption. As calcination temperature increased, the atomic fraction of C gradually decreased (from 53.65% in MBC to 30.40% in MBC700), while that of O proportion increased (from 18.61% in MBC to 38.36% in MBC500). The N proportion initially rose, reaching a maximum of 6.69% at MBC300, before declining. This trend is linked to the thermal alteration of carbonaceous matter and the simultaneous enrichment of oxygen-containing functional groups. The thermal decomposition of nitrogen-containing groups generated active sites for ENR adsorption [37].

A new F1s peak appeared near 685 eV alongside the C1s, N1s, and O1s signals after ENR adsorption (Figure 5b), confirming the successful adsorption of ENR via its fluorine-containing group. The comparative XPS analysis before and after adsorption indicated a slight decrease in C proportion and a corresponding increase in oxygen proportion. For example, the atomic fraction of C decreased from 53.65 to 52.43%, while O increased from 18.61 to 19.38% in MBC. The F proportion also exhibited a temperature-dependent trend. MBC400 exhibited the highest F proportion after adsorption (0.49 at%). Oxygen-containing functional groups such as hydroxyl and carboxyl on aerogel surface formed hydrogen bonds with ENR molecules, while nitrogen-containing groups such as amino groups contributed to π–π stacking and electrostatic interactions with ENR [42]. Additionally, the micro–mesoporous structure generated during high-temperature calcination supplied additional physical adsorption sites. MBC400 exhibited the highest ENR adsorption capacity ($Q_{e}$), owing to an optimal combination of surface functionality and pore architecture [43]. Overall, these results highlighted that calcination temperature modulated the surface chemistry and physical structure of aerogel. This control altered hydrogen bonding, π–π stacking, electrostatic interactions, and physical adsorption with ENR, ultimately determining adsorption performance.

### 2.2. Results of the Effect of Different Modified Aerogels on ENR Adsorption

**Figure 6:**
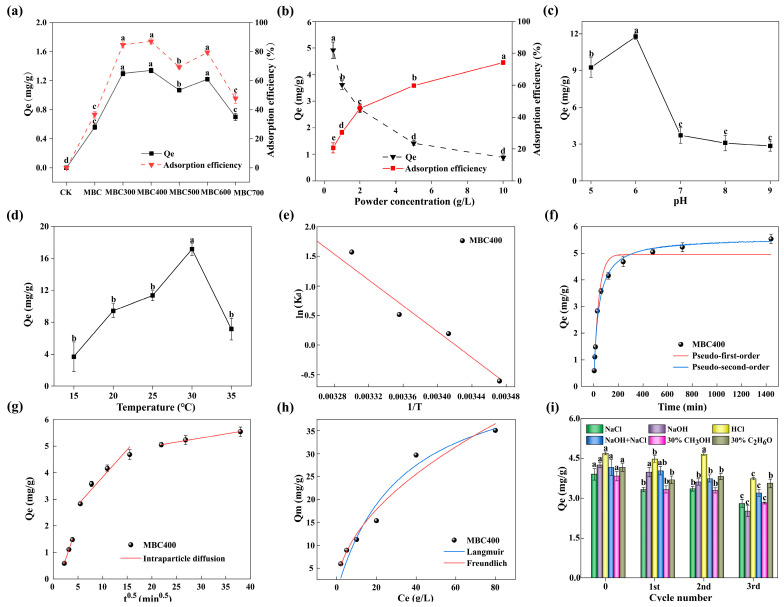
Batch result diagram. Effects of shrimp shell-derived aerogel at different calcination temperatures on ENR adsorption (**a**); the optimal dosage (**b**); effect of pH on ENR adsorption (**c**); effect of temperature on ENR adsorption (**d**); thermodynamic fit of ENR on MBC400 (**e**); adsorption kinetics (**f**,**g**); adsorption isotherms (**h**); adsorption efficiency of the six eluents (**i**). The data presented in the graph were the mean ± SD values of replicates. The different letters above the bars indicated significant differences among the different groups (*p* < 0.05, *n* = 3). Statistical significance was determined using Tukey–Kramer test. The experimental conditions were as follows: adsorbent dosage = 2 g/L, initial enrofloxacin concentration = 100 μg/L, contact time = 24 h, temperature = 25 °C, and shaking speed = 150 r/min. Other variables were adjusted according to the specific experimental conditions, as detailed in the Section 4.

For adsorption experiments, an initial ENR concentration was selected as 10 mg/L, based on a previous study [44]. Our preliminary experimental assays indicated that prolonged immersion of aquatic products at this concentration may result in body content exceeding 100 μg/kg (GB 31650–2019) [45]. This concentration provided a clear concentration-driven adsorption gradient for differentiating adsorbent performance, while also simulating environmental pollution levels of ENR to improve practical applicability. As shown in Figure 6a, both $Q_{e}$ and removal efficiency of ENR depended strongly on calcination temperature. As calcination temperature increased from 300 to 400 °C, both the $Q_{e}$ and removal efficiency of ENR increased significantly (Tukey–Kramer test, *p* < 0.05). MBC400 demonstrated the highest adsorption capacity and removal efficiency among all samples. The superior performance of MBC400 was attributable to its well-developed pore structure and abundant oxygen-containing surface groups such as carboxyl and hydroxyl groups, which provided ample active adsorption sites for ENR [45]. However, adsorption performance declined during the higher calcination temperatures (500–700 °C), likely due to the pore collapse and thermal decomposition of surface functional groups [46]. In conclusion, calcination temperature critically controlled adsorption performance of shrimp shell-derived aerogel. MBC400 was identified as a more suitable material for ENR adsorption.

### 2.3. Result of Batch Experiments

#### 2.3.1. Result of the Effect of Adsorbent Dosage on ENR Adsorption

The adsorbent dosage is one of the critical parameters influencing the ENR adsorption efficiency. To assess this effect, the impact of MBC400 adsorbent dosage on ENR adsorption was investigated under constant conditions of pH, temperature, response time, and initial ENR concentration. As shown in Figure 6b, $Q_{e}$ of ENR decreased from approximately 4.92 mg/g at a dosage 0.5 g/L to about 0.88 mg/g at 10 g/L. In contrast, adsorption efficiency exhibited a continuous upward trend, rising from 20.7% at 0.5 g/L to over 74.2% at 10 g/L. Adsorption efficiency reached a significantly (Tukey–Kramer test, *p* < 0.05) high level at a dosage of 2 g/L, accompanied by substantial ENR removal. Further increases in dose marginally improved adsorption efficiency, while $Q_{e}$ continued to decline. The available active sites per unit mass are abundant relative to the number of ENR molecules at low doses, allowing near-complete occupation with these sites [47]. Consequently, adsorption efficiency improved with increasing dosage owing to the larger total number of binding sites. However, particle aggregation and reduced effective surface area lower the number of accessible sites per unit mass at very high dosages, leading to a reduction in adsorption capacity [48]. Therefore, all subsequent experiments employed 2 g/L as adsorbent dosage.

#### 2.3.2. Result of the Effect of pH on ENR Adsorption

To investigate the effect of pH on ENR adsorption, experiments were conducted under constant parameters including adsorbent dosage, initial ENR concentration, contact time, and temperature. As illustrated in Figure 6c, $Q_{e}$ of ENR increased significantly (Tukey–Kramer test, *p* < 0.05) from approximately 9.26 mg/g at pH 5 to a maximum of 11.76 mg/g at pH 6. Beyond pH 6, $Q_{e}$ exhibited a declining trend, dropping to 3.09 mg/g at pH 8 and remaining relatively stable at pH 9. This behaviour was closely related to dissociation characteristics of ENR and surface charge properties of shrimp shell-derived aerogel. ENR possessed two specific acid dissociation constants (pK_a1_ and pK_a2_) [44], which determine its predominant ionic species at given pH values. When solution pH falls within an optimal range (e.g., around 6), the synergistic effects between ENR molecules and functional groups such as carboxyl and hydroxyl supported hydrogen bonding and electrostatic attraction, maximizing the adsorption process. In contrast, when pH departed from the optimum, ENR speciation and aerogel surface charge changed, weakening hydrogen bonds and electrostatic interactions, leading to reduced adsorption capacity [45].

#### 2.3.3. Result of the Effect of Temperature on ENR Adsorption

To evaluate the temperature dependence of ENR adsorption, adsorption experiments were conducted at different temperatures (15–35 °C). As shown in Figure 6d, $Q_{e}$ of ENR increased from approximately 3.71 mg/g at 15 °C to 11.35 mg/g at 25 °C, reaching a significant (Tukey–Kramer test, *p* < 0.05) maximum of 17.17 mg/g at 30 °C. $Q_{e}$ decreased to 7.14 mg/g when the temperature reached 35 °C. These results indicate that moderate temperature promoted the adsorption process. Furthermore, the relationship between ln(K_d_) and 1/T was fitted (Figure 6e). The thermodynamic parameters including Gibbs free energy (ΔG), enthalpy change (ΔH), and entropy change (ΔS) are listed in Table S4. ΔG became more negative with increasing temperature (e.g., ΔG = −1.280 kJ/mol at 25 °C, ΔG = −3.957 kJ/mol at 30 °C), indicating that the adsorption process was spontaneous and became thermodynamically more favourable at higher temperature [49]. The enthalpy change was positive (ΔH = 99.167 kJ/mol), confirming an endothermic adsorption process. Moreover, since this value was lower than the enthalpy change typically required for chemisorption (ΔH > 80 kJ/mol), the process was dominated by physical adsorption [50]. The large positive entropy change (ΔS = 339.199 J/(mol·K)) suggested an increase in system disorder on adsorption, which was commonly attributed to solvent release or increased freedom of the adsorbed species on the surface [51].

#### 2.3.4. Result of the Adsorption Kinetics

As evidently shown in Figure 6f, the adsorption process of ENR on MBC400 was characterized by an initial rapid uptake followed by a slower approach to equilibrium. During the first 120 min, $Q_{e}$ surged from nearly 0 mg/g to approximately 4.16 mg/g, accounting for over 65% of the ultimate equilibrium capacity. This rapid uptake can be attributed to the relatively high specific surface area and well-developed pore structure of MBC400, which provided abundant binding sites for ENR molecules and facilitated rapid attachment [52]. A notable decrease in $Q_{e}$ and rate was observed, owing to the gradual saturation of the adsorbent’s active sites. The apparent adsorption equilibrium was attained after approximately 480 min, with a final experimental equilibrium $Q_{e}$ of 5.05 mg/g.

To further examine diffusion kinetics and rate-limiting steps of ENR adsorption on MBC400, the experimental data were systematically fitted to pseudo-first-order (PFO), pseudo-second-order (PSO), and intraparticle diffusion (IPD) models. As summarized in Table S5 and Figure 6f, the PSO model produced a slightly higher correlation coefficient (*r*^2^ = 0.991) compared to the PFO model (*r*^2^ = 0.989). Moreover, the theoretical adsorption capacity calculated from the PSO model ($Q_{e}$ = 5.596 ± 0.199 mg/g) matched the experimental value. These findings indicated that ENR adsorption on MBC400 followed the PSO model more appropriately, which may imply a contribution from chemisorption such as bond formation or electron transfer between adsorbent and adsorbate [53]. The adsorption rate constant (k_2_ = 0.005 ± 0.005 g/(mg·min)) derived from the PSO model reflected the adsorption rate of ENR. The larger k_2_ values indicated a faster approach to equilibrium.

Based on fitting results of the IPD model (Figure 6g), the adsorption process exhibited clear multi-linear behaviour, indicating it was not solely controlled by intraparticle diffusion. The plot of $Q_{e}$ versus t^0.5^ revealed three distinct linear segments. First stage (0–5 min^0.5^): a rapid initial segment with a high slope, where $Q_{e}$ increased to approximately 1.51 mg/g. This stage corresponded to the rapid external surface diffusion of ENR molecules to the adsorbent surface, which was driven by a high concentration gradient. Second stage (5–20 min^0.5^): a transition segment with a moderate slope, where $Q_{e}$ increased to approximately 3.20 mg/g. This stage reflected the gradual diffusion of ENR molecules into the mesopores of the aerogel. Third stage (20–35 min^0.5^): a slower final segment with a low slope, where $Q_{e}$ rose to the final equilibrium value of 5.62 mg/g. This stage represented the intraparticle diffusion into the micropores of the aerogel. Critically, the fitted line did not pass through the origin, with an intercept of approximately 4.38 mg/g, indicating that external surface diffusion also significantly influenced the overall adsorption rate. Initially, ENR molecules rapidly diffused to MBC400 external surface, where rapid adsorption was driven by mass transfer. Intraparticle diffusion may dominate intermediate stages. However, IPD curve does not intersect the origin, suggesting that intraparticle diffusion cannot be the sole rate-limiting process. The micropore and mesoporous structure of aerogel also significantly contributed to the adsorption process [54]. ENR adsorption involved a combination of multiple processes, including external liquid-film diffusion, chemical adsorption, and surface adsorption, rather than a single adsorption mechanism [55]. Therefore, the adsorption process of ENR on MBC400 was best described as a composite process involving mass-transfer and surface-binding steps.

#### 2.3.5. Result of the Adsorption Isotherms

To investigate the adsorption isotherm characteristics of ENR, experiments were conducted under constant parameters including adsorbent dosage, pH, temperature, and contact time. The experimental data were fitted to Langmuir and Freundlich models (Figure 6h). The experimental $Q_{e}$ increased with the initial ENR concentration ($C_{e}$), rising from approximately 11.25 mg/g at $C_{e}$ = 10 g/L to 35.08 mg/g at $C_{e}\;$= 80 g/L, indicating a concentration-dependent adsorption behaviour. Freundlich model (red curve) exhibited a correlation coefficient (*r*^2^) closer to 1 compared to the Langmuir model (blue curve), demonstrating a superior agreement with the experimental data. Table S5 summarizes the parameter values goodness of fit statistics for both models. Compared with the Langmuir model, Freundlich model had a smaller Reduced Chi–Sqr and a larger (*r*^2^) and Adjusted (*r*^2^), indicating a better overall fit. These results confirmed that ENR adsorption by MBC400 can be better described by Freundlich isotherm. The adsorption process was heterogeneous multilayer adsorption, which reflected a non-uniform distribution of active sites and heterogeneous site energy on the adsorbent surface [56]. The maximum adsorption capacity ($q_{max}$) was estimated to be 14.56 mg/g, which was comparable to or higher than values reported for other carbon-based adsorbents in the literature [57,58]. This performance underscored the structural advantages of shrimp shell-derived aerogel, such as its hierarchical porosity and abundant surface functional groups, which enhanced its adsorption capability relative to unmodified agricultural waste.

#### 2.3.6. Result of the Adsorbent Regeneration and Reusability

Furthermore, adsorbents’ reusability is crucial for economic viability and resource efficiency in practical applications. This study investigated the regeneration efficiency of six eluents. As illustrated in Figure 6i, $Q_{e}$ was consistent across all eluents in the first cycle, with values ranging from approximately 3.2 to 4.5 mg/g, indicating similar initial adsorbent performance. After three regeneration cycles, most eluents exhibited declining adsorption capacities, suggesting progressive loss or blockage of active sites after multiple regeneration processes [50]. Notably, adsorbent regenerated with 30% ethanol maintained relatively high adsorption capacity of approximately 3.56 mg/g after three cycles, indicating that 30% ethanol effectively desorbed ENR and better restores active sites. In contrast, adsorbent regenerated with 1 M NaOH exhibited a more pronounced decline, dropping to approximately 2.52 mg/g after three cycles, likely due to incomplete desorption combined with potential chemical alteration or fouling of adsorption sites [59]. The remaining four eluents revealed intermediate regeneration performances, with $Q_{e}$ between 2.8 and 3.2 mg/g after three cycles. Overall, 30% ethanol demonstrated the best regeneration performance among the tested eluents, offering a more promising solution for practical recyclability. However, further optimization of the regeneration process is still needed to increase the number of viable regeneration cycles and improve efficiency while accounting for environmental impact and cost.

### 2.4. Adsorption Mechanism

**Figure 7:**
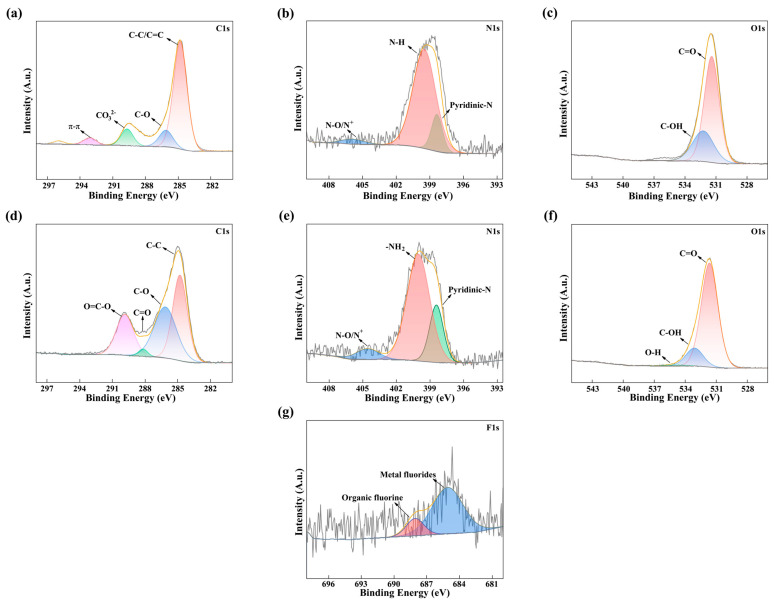
XPS peak spectra of MBC400 before ENR adsorption by C1s (**a**), N1s (**b**), O1s (**c**). Peak spectra after adsorption of ENR by C1s (**d**), N1s (**e**), O1s (**f**) and F1s (**g**).

XPS was used to explore the adsorption mechanism of ENR on shrimp shell-derived aerogel. The C1s spectra were deconvoluted into three peaks corresponding to C–C/C=C (~285 eV, graphitic/aliphatic carbon), C–O (~286.4 eV, ether/hydroxyl), and O–C=O (~288 eV, carboxyl) groups before ENR adsorption (Figure 7 and Figures S3–S7a) [60]. As calcination temperature increased, the relative intensity of the C–O component increased up to 400 °C and then declined, which was consistent with thermally induced generation of oxygenated surface groups. The proportion of C=O group increased between 500 and 700 °C, suggesting oxidative rearrangement of carbon matrix at elevated temperatures [61]. Both binding energies and relative abundances of C1s components changed after ENR adsorption (Figure 7 and Figures S3–S7d). MBC400 exhibited the largest reduction in the C–O fraction and notable shifts in O–C=O binding energy, consistent with electron-density transfer and specific interactions between ENR and surface carboxyl/hydroxyl groups. Therefore, calcination temperature influenced the interaction strength with ENR by modulating surface functional group [62].

The N1s spectra were deconvoluted into N–H (~400 eV, amine/ammonium) and N–O (~403 eV, oxidized nitrogen) components before ENR adsorption (Figure 7 and Figures S3–S7b). The proportion of N–H peaks initially increased with increasing temperature and then declined between 300 and 400 °C. A pyridinic-N component (~399 eV) gradually appeared from 500 to 700 °C, resulting from thermal formation of N-heterocycles during high-temperature pyrolysis [49]. The −NH^+^ characteristic peak was most prominent in MBC400 after ENR adsorption (Figure 7 and Figures S3–S7e), indicating stronger protonation or electrostatic interactions between amino sites and ENR molecules at this temperature. Additionally, nitrogen heterocycles generated at high temperature likely contribute to adsorption through π–π stacking with the aromatic moieties of ENR [63].

The O1s spectra were deconvoluted into C–O (~533 eV, ether/hydroxyl) and C=O (~531 eV, carbonyl) components prior to ENR adsorption (Figure 7 and Figures S3–S7c). The C–O fraction increased from 0 to 400 °C and peaked at 400 °C, indicating enrichment of hydroxyl/ether functionalities. The Ca–O component (~531 eV) attributable to CaCO_3_, became dominant while organic O-containing species diminished from 500 to 700 °C. MBC400 exhibited an increased O–H signal (~534.0 eV) with a small binding-energy downshift after ENR adsorption (Figure 7 and Figures S3–S7f), demonstrating hydrogen-bonding interactions between ENR hydroxyl groups and the aerogel surface [64]. Thus, MBC400 provided the strongest hydrogen-bonding sites. The increased proportion of inorganic oxygen functional groups such as Ca–O weakened hydrogen bonding with ENR at higher temperatures (500–700 °C) [65].

An organic F peak (~687 eV) was detected in all samples after ENR adsorption (Figure 7 and Figures S3–S7g), consistent with the fluorine-containing group in ENR. MBC400 revealed the largest F 1s intensity and additionally displayed a metal-fluoride component, suggesting partial surface complexation between shell-derived Ca^2+^ and ENR fluorine atoms (Ca–F formation) alongside incorporation of organic fluorine [66].

**Figure 8:**
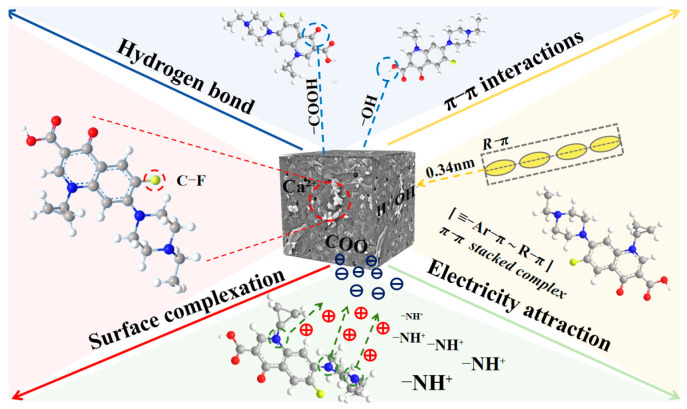
Adsorption mechanism of ENR by shrimp shell-derived aerogel.

The calcination temperature modulated the presence and relative abundance of carbon, nitrogen, and oxygen functional groups and promoted phase changes in inorganic components, thereby influencing the interaction with ENR and aerogel [67]. The abundant amino and hydroxyl groups facilitated ENR adsorption mainly through hydrogen bonding and electrostatic interactions at low temperatures (0–300 °C). At medium temperature (400 °C), synergistic effects among carboxyl, hydroxyl, and pyridinic-N groups, combined with Ca^2+^–F interactions, drove adsorption via hydrogen bonding, π–π stacking, electrostatic interactions, and surface complexation, producing optimal adsorption performance (Figure 8). The reduction in oxygen-containing organic functional groups and the increase in inorganic oxygen functional groups weaken hydrogen bonding with ENR and aerogel at high temperatures (500–700 °C). In conclusion, 400 °C was the optimal calcination temperature for ENR adsorption by shrimp shell-derived aerogel, because it maximizes the coexistence of functional groups and pore structure that promote synergistic adsorption mechanisms.

## 3. Conclusions

This study utilized *Litopenaeus vannamei* shells as raw materials to prepare a series of aerogel (MBC300–MBC700) through chemical modification followed by pyrolysis at different temperatures (300–700 °C). Based on the material characterization results, MBC400 with relatively optimal structural properties was selected for systematic investigation of its adsorption performance toward ENR. ENR adsorption on the optimal material (MBC400) fitted a pseudo-second-order kinetic model (*r*^2^ = 0.9910) and Freundlich isotherm model (*r*^2^ = 0.9614), confirming that the adsorption process was dominated by chemical adsorption and heterogeneous multilayer adsorption. The maximum adsorption capacity of MBC400 for ENR reached 14.56 mg/g under suitable conditions (dosage: 2 g/L, pH = 6, temperature: 25 °C). Thermodynamic analysis revealed that the adsorption process was spontaneous (ΔG < 0), endothermic (ΔH > 0), and associated with an increase in entropy (ΔS > 0). Regeneration tests identified 30% ethanol as the most effective eluent, and the adsorption efficiency of MBC400 remained above 75% after three adsorption–desorption cycles. MBC400 possessed a well-defined micro–mesoporous architecture rich in hydroxyl, carboxyl, and amino functional groups, providing accessible pores and active sites for the adsorption of ENR. The retention of ENR by MBC400 entailed the concerted action of several mechanisms, such as hydrogen bonding, π–π stacking, electrostatic forces, and surface complexation. This work not only offers a novel route for the high-value utilization of shrimp shell waste, but also provides a low-cost adsorbent and theoretical support for the efficient removal of fluoroquinolone antibiotics from aquaculture wastewater.

## 4. Materials and Methods

### 4.1. Materials

Aerogel was prepared using the shells of *Litopenaeus vannamei* obtained from Dongping Liuyan Aquatic Products Store (Wenzhou, China). The chemical reagents employed in the experiments, including hydrochloric acid (HCl), sodium hydroxide (NaOH, ≥98.0%), methanol (CH_3_OH, ≥99.9%), enrofloxacin (ENR, C_19_H_22_FN_3_O_3_, 98.0%), formic acid (HCOOH, ≥98.0%), acetonitrile (C_2_H_3_N, ≥99.9%), and glacial acetic acid (CH_3_COOH, ≥99.8%), were purchased from Shanghai Anpu Yun Biotechnology Co., Ltd. (Shanghai, China). All solutions were prepared using distilled water supplied by Watsons (Beijing, China). An ENR stock solution (10 mg/L) was prepared by dissolving ENR in a small amount of methanol to enhance its solubility, followed by dilution with distilled water to the different concentration.

### 4.2. Preparation of Shrimp Shell-Derived Aerogel

The shrimp shells were rinsed three times with distilled water, dried at 60 °C for 12 h, and ground into powder. The sequential chemical modification was employed to enhance the specific surface area of aerogel [68]. Our preliminary experimental results indicated that combined modification with NaOH and HCl at a 4:1 concentration ratio significantly increased the specific surface area of aerogel. The specific procedure was as follows: 10.0 g of shrimp shell was dispersed in 50 mL of 4 M NaOH solution and stirred magnetically at 60 °C for 12 h. The solid was collected by centrifugation (6000 r/min, 10 min) and washed three times with distilled water. The resulting solid was then placed in an equal volume of 1 M HCl solution and stirred at same condition. After centrifugation, the sample was washed with distilled water until the filtrate became neutral. The modified substance was lyophilized at 60 °C for 24 h, then transferred to a muffle furnace (M10L–1400, SGM, Shanghai, China). The programme was set to ramp from room temperature to target temperatures (300, 400, 500, 600, and 700 °C) at 10 °C/min, with a 120 min hold at each temperature. The resulting derived aerogel was sieved through a 200–mesh screen (0.074 mm) and labelled as MBC300, MBC400, MBC500, MBC600, and MBC700. The untreated aerogel was labelled as raw shrimp shells (MBC).

### 4.3. Characterization of Aerogel

The surface morphology and microstructure of the aerogel were characterized by field-emission scanning electron microscopy (SEM; Carl Zeiss, Merlin Compact, Schneider Electric, Neuss, Germany) [69]. A small amount of the dried aerogel was dispersed and mounted onto conductive adhesive tape, followed by a thin layer of gold under vacuum to improve surface conductivity. Secondary electron images were acquired at multiple magnifications under an accelerating voltage of 1.0–15.0 kV.

Nitrogen adsorption–desorption isotherms were measured at 77 K using an ASAP 2460 physisorption analyzer (Micromeritics, Norcross, GA, USA). Aerogel was degassed under vacuum at 200 °C for 12 h to remove adsorbed moisture and volatile impurities. The specific surface area was calculated from adsorption data in the relative pressure (P/P_0_) range of 0.05–0.30 using the BET model. The pore size distribution and total pore volume were determined from desorption branch by the Barrett–Joyner–Halenda (BJH) method.

Thermogravimetric analysis (TG) was performed using an instrument (209F3A, NETZSCH, Waldkraiburg, Germany) to evaluate the thermal stability and composition of aerogel [70]. Approximately 10 mg of aerogel was placed in an Al_2_O_3_ crucible and heated from room temperature to 800 °C at a rate of 10 °C/min under a high-purity N_2_ atmosphere. The mass loss was recorded continuously as a function of temperature.

Fourier transform infrared spectroscopy (FTIR; Perkin Eimer Frontier, Waltham, MA, USA) was employed to identify surface functional groups and chemical characteristics [71]. The dried aerogel was thoroughly mixed with spectroscopic-grade KBr and pressed into a transparent pellet for FTIR analysis. Spectra were recorded at room temperature in the wavenumber range of 4000–400 cm^–1^ with a resolution of 4 cm^–1^.

X-ray diffraction (XRD) patterns were recorded (Smart Lab 9KW, Rigaku, Tokyo, Japan) with Cu Kα radiation (λ = 0.154 nm) to analyze the crystalline phase and structure [72]. Patterns were collected from 2θ = 10° to 80° at a step size of 0.02° and room temperature. Phase identification was carried out by matching the diffraction patterns with reference data in the International Centre for Diffraction Data (ICDD) database using Jade software 9.

X-ray photoelectron spectroscopy (XPS; ESCALAB250Xi, Thermos Scientific, Waltham, MA, USA) was conducted to determine surface elemental composition and chemical states [73]. A monochromatic Al Kαsource (1486.6 eV) was used. The binding energy scale was calibrated to the C 1s peak of adventitious carbon at 284.8 eV. Data processing and peak fitting were performed using the Avantage Software 2024 (Thermos Scientific, Waltham, MA, USA). The chromatographic conditions employed a Waters ACQUITY UPLC BEH C18 column (100 mm × 2.1 mm, 1.7 μm), with the column temperature maintained at 30 °C. The mobile phase consisted of an aqueous solution containing 0.1% formic acid (A) and a mixed solvent of methanol/acetonitrile/0.1% formic acid (2:8:0.1, *v*/*v*) (B). A gradient elution program was employed as detailed in Table S1, with a flow rate of 0.4 mL/min and an injection volume of 10 μL. The mass spectrometer employed an electrospray ionization (ESI) source operating in positive ion mode. Detection utilized multiple reaction monitoring (MRM) mode. Key source parameters were optimized as follows: spray voltage, 3.5 kV; desolation gas temperature, 450 °C; desolation gas flow rate, 50 L/h; ion source temperature, 150 °C; and collision energy, 3 V. Collision gas consisted of high–purity argon (≥99.99%) at a pressure of 1.5 mTorr. Parent ions, qualitative ions, quantitative ions, cone voltage, and collision energy are summarized in Table S2.

### 4.4. Batch Experiments

#### 4.4.1. Effect of Different Modified Aerogel on ENR Adsorption

A 100 mL of 10 mg/L ENR solution was prepared in a 250 mL conical flask. For each flask, 1.0 g of aerogel (MBC, MBC300, MBC400, MBC500, MBC600, and MBC700) was added, and the initial pH was adjusted to 5.0. The mixtures were shaken in a constant-temperature incubator shaker at 25 °C and 150 rpm for 24 h to allow dynamic adsorption. All experimental groups were performed in triplicate under identical conditions.

After adsorption, 5 mL of supernatant was transferred into a 50 mL centrifuge tube. Then, 50 µL of ENR–D5 internal standard solution and 5 mL of 1% acetic acid in acetonitrile were added. The mixture was vortexed at 2500 r/min for 10 min and subsequently centrifuged at 4500× *g* for 10 min. The supernatant was collected and evaporated to near dryness under a N_2_ at 40 °C. The residue was reconstituted in 1 mL of 0.1% formic acid in water and vortexed at 2500 r/min for 10 min. The resulting solution was filtered through a 0.22 μm aqueous phase filter membrane and transferred into an autosampler vial for analysis. ENR concentration was determined using high-performance liquid chromatography (HPLC; TSQ Quantum Access Max, Thermos Scientific, Waltham, MA, USA).

#### 4.4.2. Effect of Adsorbent Dosage on ENR Adsorption

A 100 mL of 10 mg/L ENR solution was prepared in a 250 mL conical flask. MBC400 was added at dosage of 0.05 g (0.5 g/L), 0.10 g (1 g/L), 0.20 g (2 g/L), 0.50 g (5 g/L), and 1.00 g (10 g/L), respectively. The initial pH was adjusted to 5.0. The mixtures were shaken in a constant-temperature incubator shaker at 25 °C and 150 rpm for 24 h to perform dynamic adsorption. All experimental groups were performed in triplicate under identical conditions. Water sample pretreatment method followed the procedure described in Section 4.4.1.

#### 4.4.3. Effect of pH on ENR Adsorption

The 0.2 g of MBC400 was added to 100 mL of 10 mg/L ENR solution. Under constant conditions (25 °C, 150 rpm, 24 h), the adsorption process was evaluated at varying initial pH levels of 5.0, 6.0, 7.0, 8.0, and 9.0. All experiments were carried out in triplicate under identical conditions. The water sample pretreatment method was the same as described in Section 4.4.1.

#### 4.4.4. Effect of Temperature on ENR Adsorption

The 0.2 g of MBC400 was added to 100 mL of 10 mg/L ENR solution. The temperatures were set to 15, 20, 25, 30, and 35 °C, respectively, for dynamic adsorption at 150 rpm for 24 h. All experiments were performed in triplicate under identical conditions. The water sample pretreatment method was the same as described in Section 4.4.1.

### 4.5. Adsorption Kinetics

A 100 mL of 10 mg/L ENR solution was prepared in a 250 mL conical flask, and 0.2 g of MBC400 was added. The initial pH was adjusted to 5.0. The mixture was shaken in a constant-temperature incubator shaker at 25 °C and 150 rpm for 24 h to perform dynamic adsorption. All experiments were performed in triplicate under identical conditions. Water samples (5 mL) were collected at reaction times of 0, 5, 10, 15, 30, 60, 120, 240, 480, 720, and 1440 min for analysis. The water sample pretreatment method was the same as described in Section 4.4.1. The experimental data were fitted using the pseudo-first-order kinetic model (Equation (1)) and pseudo-second-order kinetic model (Equation (2)):

(1) $$ln(q_{e}-q_{t})=lnq_{e}-k_{1}t$$

(2) $$q_{t}=k_{2}q_{e}^{2}t/(1+k_{2}q_{e}t)$$

where $q_{e}$ was adsorption capacity of aerogel for ENR at equilibrium (mg/g), $q_{t}$ was adsorption capacity of aerogel for ENR at time t (mg/g), $k_{1}$ was rate constant of the pseudo-first-order kinetics (min^–1^), $k_{2}$ was rate constant of the pseudo-second-order kinetics g/(mg·min).

### 4.6. Adsorption Isotherms

The initial ENR concentrations were set at 1, 2, 5, 10, 20, 40, and 80 mg/L. For the equilibrium isotherm experiments, 0.2 g of MBC400 was weighed into 250 mL conical flasks, and 100 mL of various ENR solutions were then added to each flask. The flasks were placed in a constant-temperature incubator shaker for dynamic adsorption at 25 °C and 150 rpm for 24 h. All isotherm experiments were performed in triplicate under identical conditions. Water sample pretreatment method was the same as described in Section 4.4.1. The experimental data were fitted using the Langmuir (Equation (3)) and Freundlich (Equation (4)) isotherm models:

(3) $$q_{e}=q_{m}K_{L}c_{e}/(1+K_{L}c_{e})$$

(4) $$q_{e}=K_{F}c_{e}^{1/m}$$

where $q_{m}$ was maximum theoretical adsorption capacity (mg/g), $c_{e}$ was equilibrium concentration of ENR (mg/L), $K_{L}$ was Langmuir model constant (L/mg), $K_{F}$ was Freundlich model constant, 1/m was Freundlich model constant.

### 4.7. Adsorbent Regeneration and Reusability

Under optimal adsorption conditions, ENR-loaded MBC400 was transferred to 50 mL centrifuge tubes and centrifuged at 8000× *g* for 5 min to collect the precipitate. Six different eluents (30 mL each of 0.5 mol/L NaCl, 2 mol/L NaOH, 1 mol/L HCl, 10% NaOH + 5% NaCl, 30% methanol, and 30% ethanol) were used for desorption treatment. The precipitate was resuspended in eluent, vortexed for 2 min, sonicated for 30 min, and centrifuged at 8000× *g* for 5 min. The regenerated adsorbent was then collected and dried at 60 °C for 12 h. The regenerated adsorbent was reused in subsequent adsorption experiments. Three adsorption–desorption cycles were consecutively performed. The adsorption experiments followed the procedure described in Section 4.4.1. ENR adsorption capacity and removal efficiency were measured after each cycle to compare the regeneration effectiveness of the different desorption methods [74].

### 4.8. Statistical Analysis

Statistical analysis was conducted with SPSS 26.0, while data visualization was carried out using Origin 2025. Statistical significance was assessed using Student’s *t*-test and Analysis of Variance (ANOVA), followed by Tukey–Kramer test in case of significant effect. Normality and homogeneity of variance were checked prior to the ANOVA analysis by Kolmogorov–Smirnov’s and Levene’s tests, respectively. Statistical significance was defined as *p* < 0.05. All data, collected from a minimum of three independent experiments, are expressed as mean ± standard deviation.

## Data Availability

The original contributions presented in this study are included in the article. Further inquiries can be directed to the corresponding author.

## Supplementary Materials

The following supporting information can be downloaded at: https://www.mdpi.com/article/10.3390/gels12030247/s1. Figure S1: Isotherm linear Plot of MBC (a); MBC300 (b); MBC400 (c); MBC500 (d); MBC600 (e); MBC700 (f); Figure S2: Isotherm log plot of MBC (a); MBC300 (b); MBC400 (c); MBC500 (d); MBC600 (e); MBC700 (f). Figure S3: XPS peak spectra of MBC before ENR adsorption by C1s (a), N1s (b), O1s (c). Peak spectra after adsorption of ENR by C1s (d), N1s (e), O1s (f) and F1s (g). Figure S4: XPS peak spectra of MBC300 before ENR adsorption by C1s (a), N1s (b), O1s (c). Peak spectra after adsorption of ENR by C1s (d), N1s (e), O1s (f) and F1s (g). Figure S5: XPS peak spectra of MBC500 before ENR adsorption by C1s (a), N1s (b), O1s (c). Peak spectra after adsorption of ENR by C1s (d), N1s (e), O1s (f) and F1s (g). Figure S6: XPS peak spectra of MBC600 before ENR adsorption by C1s (a), N1s (b), O1s (c). Peak spectra after adsorption of ENR by C1s (d), N1s (e), O1s (f) and F1s (g). Figure S7: XPS peak spectra of MBC700 before ENR adsorption by C1s (a), N1s (b), O1s (c). Peak spectra after adsorption of ENR by C1s (d), N1s (e), O1s (f) and F1s (g). Table S1: Liquid-phase elution conditions. Table S2: Parent ions, qualitative ions, quantitative ions, cone voltage, and collision energy for ENR and ENR–D5. Table S3: The surface properties of MBC and MBC300–MBC700 were calculated from nitrogen adsorption–desorption isotherms. Table S4: Thermodynamic parameters of MBC400 for ENR adsorption. Table S5: Kinetic parameters of pseudo-first-order and pseudo-second-order for ENR adsorbed on MBC400. Table S6: The relevant parameters of Langmuir and Freundlich isotherm model for ENR adsorption using MBC400.
