# Supplementary material for: Valorized Shrimp Shell-Derived Aerogel for Trace Enrofloxacin Removal from Aquaculture Wastewater: Adsorption Performance and Mechanisms Exploration
Source: Gels. 2026 Mar 15;12(3):247. doi: 10.3390/gels12030247 (PMC13025983; doi:10.3390/gels12030247)
Supplement: Supplementary file 1 [file gels-12-00247-s001.zip › gels-4160583-supplementary.pdf]

[Supplementary information]

Article

Valorized Shrimp Shell-Derived Aerogel for Trace Enrofloxacin Removal from Aquaculture Wastewater: Adsorption Performance and Mechanisms Exploration

Chengci Liu [1,†], Lei Huang [1,†], Sihan Wei [1], Bohao Qi [2], Jinhua Xu [2], Xiaodong Xu [2], Lu Qiao [2,3], Zhen Yang [2], Yuanyuan Ren [2], Jincheng Li [2], Yingchun Mu [2], Mutai Bao [4,5], Meitong Li [1], Zhiyang Zhao [6,*], Xin Hu [2,*]

[1] Tianjin Key Laboratory of Organic Solar Cells and Photochemical Conversion, College of Chemistry and Chemical Engineering, Tianjin University of Technology, Tianjin 300384, China; chengci1223@stud.tjut.edu.cn (C.L.); huanglei@tjut.edu.cn (L.H.); yj20251014@stud.tjut.edu.cn (S.W.); tjutlmt@email.tjut.edu.cn (M.L.)
[2] Chinese Academy of Fishery Sciences, Beijing 100141, China; qibohao@cafs.ac.cn (B.Q.); xujh@cafs.ac.cn (J.X.); xuxiaodong@cafs.ac.cn (X.X.); qiaolu@cafs.ac.cn (L.Q.); yangzhen@cafs.ac.cn (Z.Y.); renyuany@cafs.ac.cn (Y.R.); lijc@cafs.ac.cn (J.L.); muyc@cafs.ac.cn (Y.M.)
[3] Hainan Fisheries Innovation Research Institute, Chinese Academy of Fishery Sciences, Sanya 572000, China
[4] Key Laboratory of Marine Chemistry Theory and Technology Ministry of Education, Frontiers Science Center for Deep Ocean Multispheres and Earth System, Ocean University of China, Qingdao 266100, China; mtbao@ouc.edu.cn
[5] College of Chemistry & Chemical Engineering, Ocean University of China, Qingdao 266100, China
[6] School of Chemistry and Materials Science, Nanjing University of Information Science & Technology, Nanjing 210044, China
* Correspondence: zhzh@nuist.edu.cn (Z.Z.); huxin@cafs.ac.cn (X.H.)
† These authors contributed equally to this work.

16 Pages including cover page

7 Figures

6 Tables

List of Figures and Tables

Figure S1. Isotherm linear Plot of MBC (a); MBC300 (b); MBC400 (c); MBC500 (d); MBC600 (e); MBC700 (f).

Figure S2. Isotherm log plot of MBC (a); MBC300 (b); MBC400 (c); MBC500 (d); MBC600 (e); MBC700 (f).

Figure S3. XPS peak spectra of MBC before ENR adsorption by C1s (a), N1s (b), O1s (c). Peak spectra after adsorption of ENR by C1s (d), N1s (e), O1s (f) and F1s (g).

Figure S4. XPS peak spectra of MBC300 before ENR adsorption by C1s (a), N1s (b), O1s (c). Peak spectra after adsorption of ENR by C1s (d), N1s (e), O1s (f) and F1s (g).

Figure S5. XPS peak spectra of MBC500 before ENR adsorption by C1s (a), N1s (b), O1s (c). Peak spectra after adsorption of ENR by C1s (d), N1s (e), O1s (f) and F1s (g).

Figure S6. XPS peak spectra of MBC600 before ENR adsorption by C1s (a), N1s (b), O1s (c). Peak spectra after adsorption of ENR by C1s (d), N1s (e), O1s (f) and F1s (g).

Figure S7. XPS peak spectra of MBC700 before ENR adsorption by C1s (a), N1s (b), O1s (c). Peak spectra after adsorption of ENR by C1s (d), N1s (e), O1s (f) and F1s (g).

Table S1. Liquid–phase elution conditions.

Table S2. Parent ions, qualitative ions, quantitative ions, cone voltage, and collision energy for ENR and ENR–D5.

Table S3. The surface properties of MBC and MBC300–MBC700 were calculated from nitrogen adsorption–desorption isotherms.

Table S4. Thermodynamic parameters of MBC400 for ENR adsorption.

Table S5. Kinetic parameters of pseudo–first–order and pseudo–second–order for ENR adsorbed on MBC400.

Table S6. The relevant parameters of Langmuir and Freundlich isotherm model for ENR adsorption using MBC400.

Figure S1. Isotherm linear Plot of MBC (a); MBC300 (b); MBC400 (c); MBC500 (d); MBC600 (e); MBC700 (f).

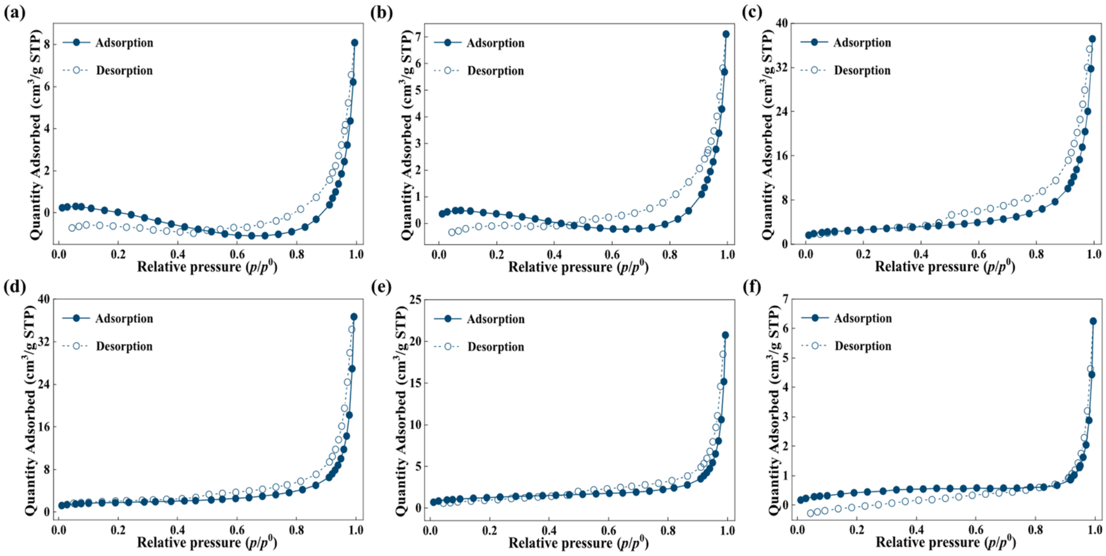

Figure S2. Isotherm log plot of MBC (a); MBC300 (b); MBC400 (c); MBC500 (d); MBC600 (e); MBC700 (f).

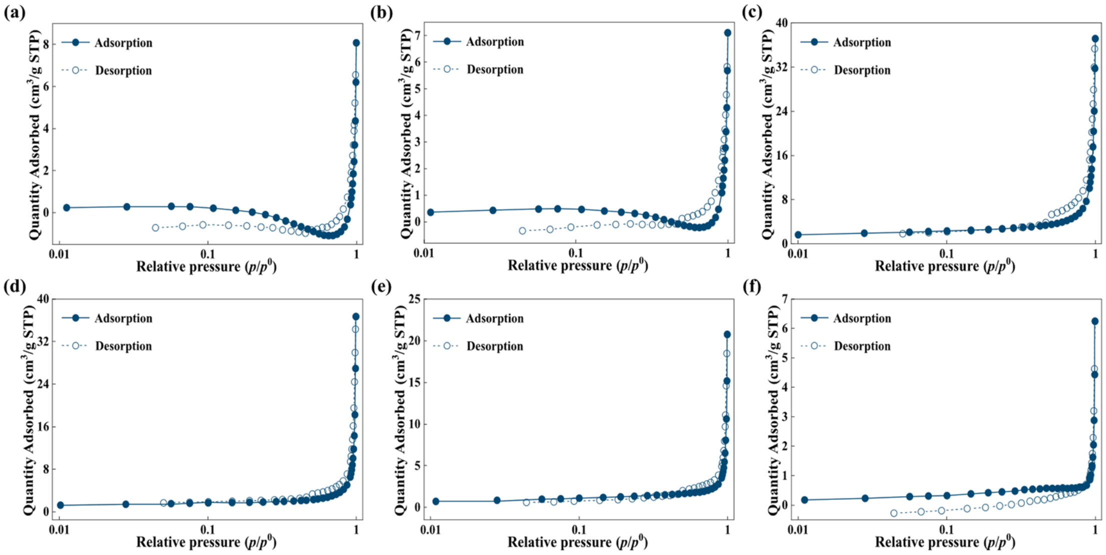

Figure S3. XPS peak spectra of MBC before ENR adsorption by C1s (a), N1s (b), O1s (c). Peak spectra after adsorption of ENR by C1s (d), N1s (e), O1s (f) and F1s (g).

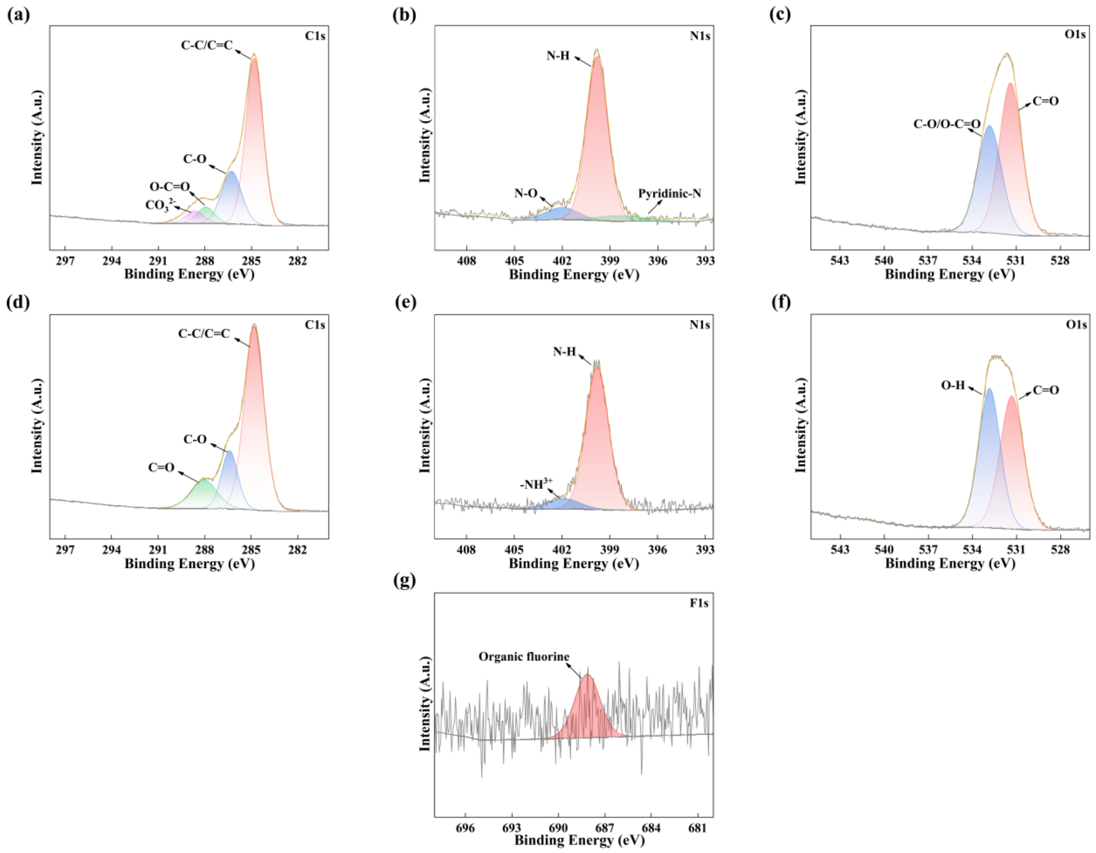

Figure S4. XPS peak spectra of MBC300 before ENR adsorption by C1s (a), N1s (b), O1s (c). Peak spectra after adsorption of ENR by C1s (d), N1s (e), O1s (f) and F1s (g).

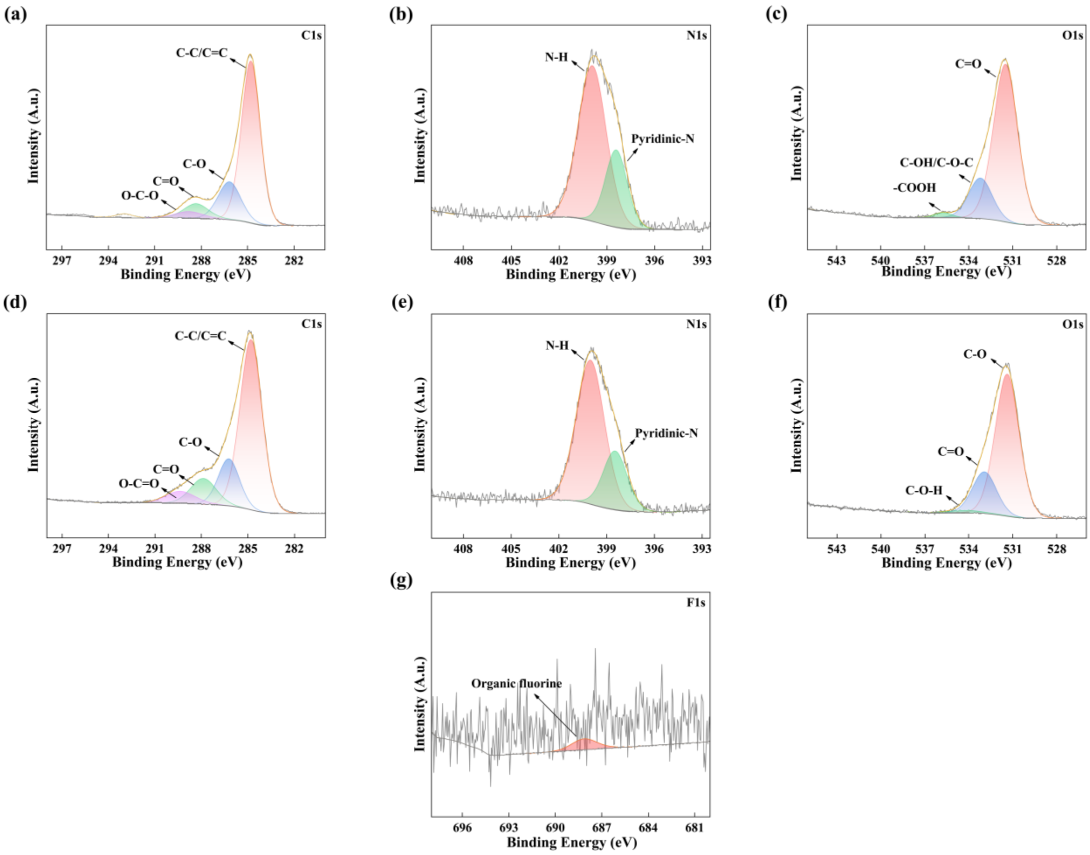

Figure S5. XPS peak spectra of MBC500 before ENR adsorption by C1s (a), N1s (b), O1s (c). Peak spectra after adsorption of ENR by C1s (d), N1s (e), O1s (f) and F1s (g).

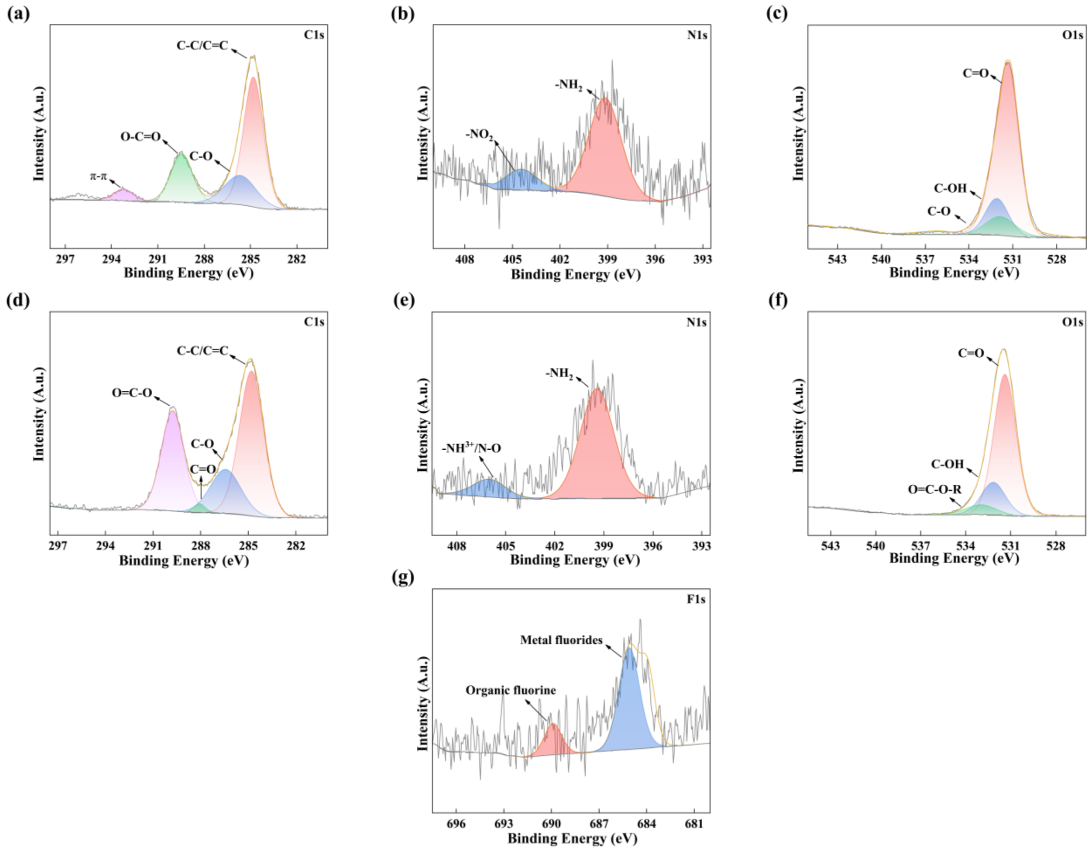

Figure S6. XPS peak spectra of MBC600 before ENR adsorption by C1s (a), N1s (b), O1s (c). Peak spectra after adsorption of ENR by C1s (d), N1s (e), O1s (f) and F1s (g).

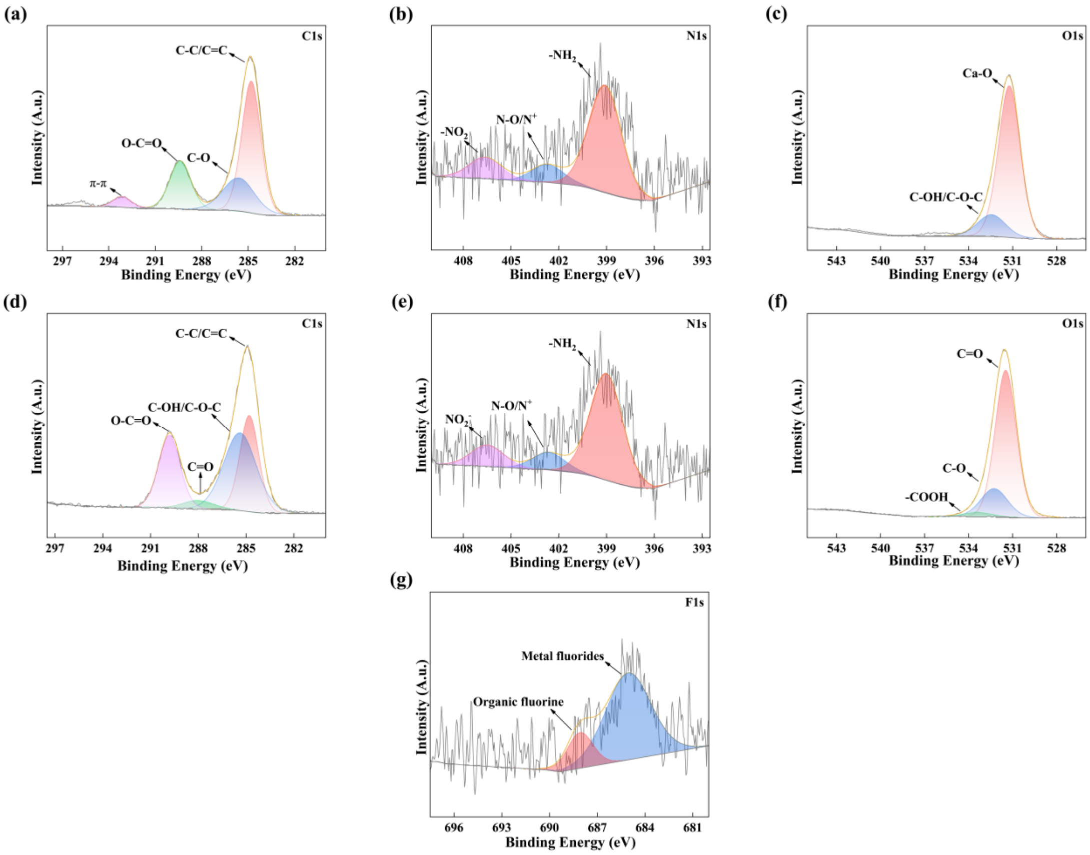

Figure S7. XPS peak spectra of MBC700 before ENR adsorption by C1s (a), N1s (b), O1s (c). Peak spectra after adsorption of ENR by C1s (d), N1s (e), O1s (f) and F1s (g).

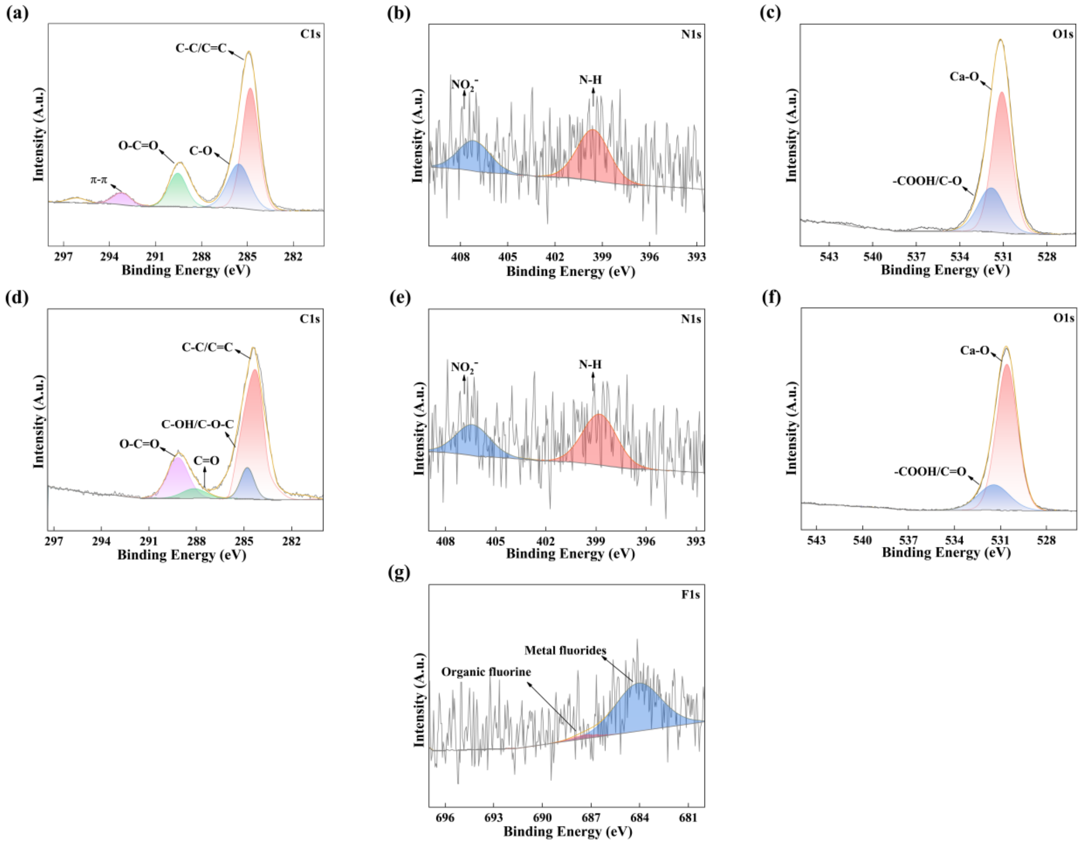

Table S1. Liquid–phase elution conditions.

Time (min)	Mobile Phase A (%)	Mobile Phase B (%)
Initial	95	5
1	95	5
3.5	90	10
13	55	45
17	30	70

Table S2. Parent ions, qualitative ions, quantitative ions, cone voltage, and collision energy for ENR and ENR–D5.

Compound	Parent ion (m/z)	Daughter ion (m/z)	Cone voltage (V)	Collision energy (V)
ENR	360.01	72.95	32	22
		84.90		16
ENR–D5	364.50	320.50	110	25
		364.50		21

Table S3. The surface properties of MBC and MBC300–MBC700 were calculated from nitrogen adsorption–desorption isotherms.

Sample	Surface area, ABET (m^2/g)	Total pore volume, V_{total} (cm^3/g)	Average pore diameter (nm)
MBC	4.3605	0.0219	40.0842
MBC300	23.6353	0.1290	15.5733
MBC400	77.7090	0.1972	9.0473
MBC500	62.4027	0.1541	8.7741
MBC600	57.2715	0.1804	10.6717
MBC700	52.1045	0.2250	12.9578

Table S4. Thermodynamic parameters of MBC400 for ENR adsorption.

Aerogel	T (℃)	r^2	ΔG (kJ/mol)	ΔH (kJ/mol)	ΔS (J/(mol·K))
	15		1.443		
	20		–0.471		
MBC400	25	0.941	–1.280	99.167	339.199
	30		–3.957		
	35		–0.948		

Table S5. Kinetic parameters of pseudo–first–order and pseudo–second–order for ENR adsorbed on MBC400.

Kinetic models	Parameters	MBC400
	Q_e (mg/g)	4.960±0.159
pseudo–first–order	k_1 (min^{-1})	0.025±0.002
	r^2	0.989
	Q_e(mg/g)	5.596±0.199
pseudo–second–order	k_2(g/(mg·min))	0.005±0.005
	r^2	0.991

Table S6. The relevant parameters of Langmuir and Freundlich isotherm model for ENR adsorption using MBC400.

**Model**	**Reduced Chi–Sqr**	**r^2 (COD)**	**Adjusted r^2**
Langmuir	8.2198	0.9535	0.9419
Freundlich	6.8335	0.9614	0.9517
